# Thalamic metabolite changes after subthalamic nucleus deep brain stimulation in Parkinson’s disease: an exploratory magnetic resonance spectroscopy study

**DOI:** 10.3389/fneur.2025.1662142

**Published:** 2025-12-03

**Authors:** Nathanael Göransson, Sofie Tapper, Peter Lundberg, Peter Zsigmond, Anders Tisell

**Affiliations:** 1 Department of Biomedical Engineering, Linköping University, Linköping, Sweden; 2 Department of Biomedical and Clinical Sciences, Linköping University, Linköping, Sweden; 3 Clinical Department of Medical Radiation Physics, Linköping University, Linköping, Sweden; 4 Department of Health, Medicine and Caring Sciences, Linköping University, Linköping, Sweden; 5 Center for Medical Image Science and Visualization (CMIV), Linköping University, Linköping, Sweden; 6 Clinical Department of Radiology, Linköping University, Linköping, Sweden

**Keywords:** deep brain stimulation, Parkinson disease, magnetic resonance spectroscopy, functional neurosurgery, movement disorder surgery

## Abstract

**Introduction:**

The mechanisms underlying the clinical effect of Deep Brain Stimulation (DBS) for Parkinson’s disease (PD) remain debated. Proton magnetic resonance spectroscopy (^1^H-MRS) provides a biochemical non-invasive *in vivo* insight. This article aims to increase the understanding of advanced PD pathophysiology and DBS using MRS before and after surgery.

**Methods:**

Eleven PD patients and seven healthy controls were included. Preoperatively and approximately 7 months postoperatively, single-voxel MRS using a PRESS sequence was performed on a 1.5 T (tesla) system. Voxels were placed bilaterally in the thalamus (14 × 13 × 13 mm^3^) and the lentiform nucleus (15 × 13 × 12 mm^3^). Metabolite concentrations of total N-acetylaspartate + N-acetyl-aspartyl-glutamate (tNA), total creatine + phosphocreatine (tCr), total choline + phosphocholine + glycerophosphocholine (tCho), and total glutamate and glutamine, which together constitute Glx were quantified. To assess treatment outcomes following surgery, medications were converted to levodopa equivalent doses (LED) using a standardized conversion formula, both pre- and post-DBS.

**Results:**

A total of 11 patients, with a mean PD duration of 9.4 years, were implanted with bilateral implantation (22 leads). All patients self-reported relief of symptoms and significantly reduced their medication (*p* < 0.001), with a calculated preoperative LED of 925 ± 272 and a postoperative LED of 611 ± 210 (mean ± SD), representing a 32% reduction after surgery. The patients, prior to surgery, compared to a healthy control group, showed no differences in the resulting metabolite concentrations (tCr, tNA, tCho, Glx) in voxels placed in the thalamus and lentiform nucleus. However, thalamic tNA concentrations differed significantly following DBS targeting the subthalamic nucleus (STN), both in comparison to healthy controls (*p* = 0.02) and relative to preoperative concentrations within the patient group (*p* = 0.03). No furher resulting concentrations differed.

**Conclusion:**

We present novel metabolite observations obtained through MRS in this exploratory study. Thalamic tNA concentrations in PD patients were comparable to those of healthy controls prior to surgery, but were significantly reduced following DBS implantation targeting the STN. These findings suggests the presence of a metabolite thalamic effect associated with DBS treatment.

## Introduction

Parkinson’s disease (PD), characterized with both motor and non- motor symptoms, is currently the second most prevalent neurodegenerative disorder (1) and the fastest-growing neurological disease, with the number of affected individuals projected to double in the coming decades (2). The pathological hallmark of PD is the loss of dopaminergic neurons in the substantia nigra pars compacta (3), and this altered innervation in the striatum results in an imbalance of activity within the basal ganglia, affecting the thalamus (1). Disruption in motor control leads to the cardinal symptoms of PD, namely bradykinesia, akinesia, rigidity, and tremor.

Disabling motor fluctuations, which can develop after an initial adequate response to levodopa, pose a significant challenge in the management of PD. (4) Deep brain stimulation (DBS) presents a safe, reversible, and adaptable therapeutic option (1), which has been demonstrated to be highly effective in managing PD symptoms (5, 6).

Despite being successfully used for almost 40 years (7), the precise mechanism by which DBS applied in diverse targets of the brain alleviates symptoms remains incompletely understood and is subjected to ongoing debate (8–12). DBS appears to act not only locally within the target area but also cause widespread network effects (9). The most common indication is PD, with the subthalamic nucleus (STN) being the preferred target (1). The STN have extensive connections with cortical and subcortical networks (9), including the lentiform nucleus (comprising the putamen and globus pallidus) of the basal ganglia (11). The Zona incerta (Zi), also having substantial connections in the brain, is located dorsally/dorsomedially to the STN, and has been explored as a target for DBS in PD (13). It may primarily be considered for tremor-dominant PD patients (14).

Proton magnetic resonance spectroscopy (^1^H-MRS) provides a biochemical *in vivo* insight into the brain. This non-invasive technique enables the acquisition of various metabolites, revealing alterations in concentrations that may underlie, cause, or be associated with neurological diseases, thereby supporting clinical decision-making (15, 16). MRS enables simultaneous quantification of several metabolites (15) that have diverse biochemical functions and may be interconnected within metabolic pathways in the brain (17). The metabolites include total creatine (tCr, originating from both creatine and phosphocreatine), total choline (tCho, consisting of phosphocholine, glycerophosphocholine, and free choline), total N-acetylaspartate and N-acetylaspartylglutamate making (tNA), and total glutamate (Glu) and glutamine (Gln) which together constitute (Glx).

In the brain, tCr is involved in cell energy homeostasis, while tCho signals membrane turnover and variations in cell density (17). tNA is primarily a neuron-specific metabolite (18) and may reflect the integrity of neuronal mitochondrial metabolism via NAA and subsequent neuronal metabolic activity (17). In addition, tNA includes NAAG, a regulatory neurotransmitter involved in modulating glutamatergic transmission (19). Finally, Glx comprises Glu, which functions as an excitatory neurotransmitter in the brain (17).

Several MRS studies have examined PD patients, revealing inconsistent metabolite findings in the subcortical structures (20). In the lentiform nucleus of PD patients, increased Cho/Cr ratios and reduced NAA/Cho have been reported (21). In contrast, other studies have found normal metabolite levels (22–25). Similarly, findings in the thalamus are inconsistent. Metabolite alterations in tremor-dominant PD, including decreased NAA/Cr and Cho/Cr ratios, have been described (26), whereas others have observed normal thalamic metabolite levels (23, 27). Finally, a previous study reported a reduction in thalamic tNA following DBS implantation in the ventral intermediate nucleus of the thalamus (VIM) for essential tremor (ET) (28), making the thalamus a region of particular interest for analysis in the context of DBS.

The primary aim of this study was to investigate metabolite concentrations in subcortical regions potentially connected to PD pathophysiology, using MRS in the thalamus and lentiform nucleus before and after DBS in a highly selective PD group. This research is novel and seeks to enhance the understanding of advanced PD pathophysiology and the underlying mechanisms of DBS.

## Materials and methods

### Patients

All patients who were diagnosed and referred by a movement disorder specialist and considered eligible for surgery were included in this prospective monocentric exploratory study. In total, 11 PD patients scheduled for DBS surgery were included with a median age of 64 years, range [56–68, Male/Female (9/2)]. All patients accepted for DBS surgery were diagnosed with idiopathic PD presenting “on–off” fluctuations with shortened “on” time and satisfactory L-dopa responsiveness. They were referred to the neurosurgical department by a senior movement disorder specialist between 2014 and 2016. All patients received symptomatic relief with anti-Parkinson medications during the MRS examination both before and after surgery. Since the patients were on several different pharmaceutical preparations, the medications were converted to *levodopa equivalent dose* (LED) using an LED conversion formula (29). The presented LED value represents the dose calculated at the time of the MRS examination. The control group consisted of seven healthy controls (HCs), median age 66 years [range 42–77; Male/Female (6/1)]. All subjects were Caucasian and right-handed. Description of the PD patients with age, sex, disease duration, and medication is summarized in Table 1 and the HC group is described in the Supplementary Table 1.

**Table 1:** Description of the PD patients.

Patient	Age	Sex	Disease duration	Pre OP LED^*^ [mg]	Post OP LED^*^ [mg]	
1	62	M	13	1,425	1,075	
2	64	M	8	1,075	450	
3^**^	64	M	12	575	550	
4^**^	68	F	9	600	600	
5	65	M	9	1,075	550	
6	61	M	12	1,025	850	
7	63	M	8	925	450	
8	62	M	9	975	700	
9	56	M	6	925	650	
10	65	M	7	500	300	
11	65	F	10	1,075	550	
Median	64	Mean	9	925	611	*p* < 0.001

Age, sex, disease duration and medication. The patients significantly reduced their medication after surgery, with a 32% reduction on group level.

* LED, Levodopa Equivalent Dose.

** Patients with leads in the zona incerta (Zi); all remaining leads were placed in the subthalamic nucleus (STN).

### Ethics

The local ethics committee at the University Hospital in Linköping (Ref. No. 2013/ 403–31, P. Zsigmond) approved the study. All subjects (patients and healthy controls) gave their written consent after receiving information about the study.

### Surgery

The T2-weighted magnetic resonance images (MRI) were used in SurgiPlan (Elekta Instruments AB, Stockholm, Sweden) for planning the direct targeting of the STN or Zi. The DBS equipment implanted was manufactured by Medtronic (Minneapolis, MN, USA), consisting of the electrode lead 3389 with extension kit 37086 and the Activa PC 37601 implantable pulse generator. A description of the surgical procedure can be found in a previous study (30).

### Magnetic resonance measurements

MR examinations were performed preoperatively and 7.0 months (range 5.1–9.1) after surgery using a 1.5 T Achieva dStream MR scanner (Philips Healthcare, Best, The Netherlands), with a Transmit/Receive head coil (as specified by the MR conditions in the instructions for use when imaging with implant). The DBS system was turned off immediately before the postoperative MRS, which was then performed within minutes. The patients were ON medication at the time of investigation and they were instructed to remain still throughout the MRS examination.

Single voxel MRS was performed using PRESS (Point REsolved Spectroscopy), an acquisition method for spatially positioning and defining a volume of interest (VOI). The following sequence settings were applied; echo time (t_TE_) 25 ms, repetition time (t_TR_) 3 s, 1 kHz sampling frequency, 128 number of water-suppressed transients were averaged. The excitation method was used for water suppression, and eight unsuppressed water transients were acquired as a reference signal. The VOIs were planned using T2w turbo spin echo sequences (TSE), the voxel was positioned with focus on the tNA-resonance chemical shift. Axial; TSE factor 15, reconstructed resolution 0.65 × 0.65 × 2 mm^3^, Field of View (FOV) 250x190x168 mm^3^, t_TE_ 80 ms, t_TR_ 8 s. Sagittal; TSE factor 15, reconstructed resolution 0.9 × 0.9 × 2 mm^3^, FOV 230x230x66 mm^3^, t_TE_ 110 ms, t_TR_ 8 s. Coronal; TSE factor 23, reconstructed resolution 0.9*0.9*2 mm^3^, (FOV) 230*183*72 mm^3^, t_TE_ 110 ms, t_TR_ 4.3 s. The technical quality of the spectra was assessed by an experienced MR expert.

**Figure 1:**
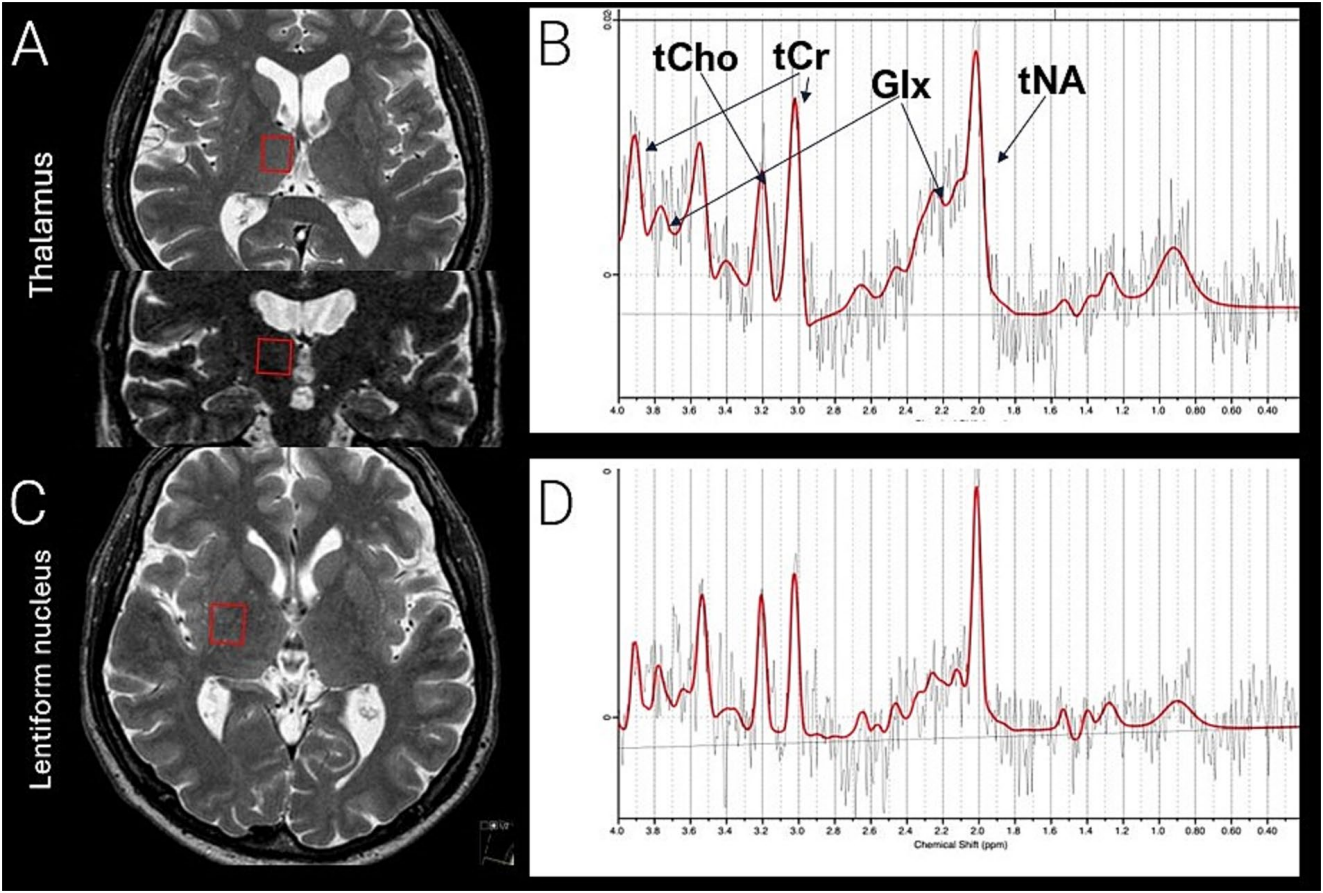
Voxel placement from the right thalamus **(A)**, seen in axial and coronal view, corresponding thalamic spectra **(B)**, right lentiform nucleus **(C)**, axial view, corresponding spectra lentiform nucleus **(D)**. tCho (total choline, phosphocholine, and glycerophosphocholine) tCr (total creatine and phosphocreatine) Glx; (total glutamate and glutamine) and tNA (total N-acetylaspartate and N-acetyl-aspartyl-glutamate). A preoperative examination is presented. In the postoperative examination, voxel placement was repeated to match the preoperative placement. Symmetrical positioning on the contralateral side was performed in each examination. Comparable voxel placement in healthy control, as presented in the figure.

A total of four MRS-voxels were placed bilaterally in the thalamus (14 × 13 × 13 mm^3^) and the lentiform nucleus (15 × 13 × 12 mm^3^) for each examination. Two trained neurosurgeons (N. G. and P. Z.) placed the voxels to ensure a comparable and reproducible location in the anatomy (Figure 1). The thalamus voxel was positioned in the superior/medial/anterior region of the thalamus. This placement focused on the motor part of the thalamus, excluded cerebrospinal fluid, and minimized the influence of adjacent anatomical structures. The voxel representing the lentiform nucleus was placed laterally to the thalamus voxel, ensuring no overlap, with the anterior part of the voxel positioned at the posterior portion of the putamen, also including the globus pallidus (Figure 1). The postoperative voxels were placed in the corresponding anatomical location as the preoperative voxels, with placement following the preoperative positioning and guided by preoperative screenshots.

Metabolite concentrations of total N-acetylaspartate and N-acetyl-aspartyl-glutamate (tNA), total creatine and phosphocreatine (tCr), total choline, phosphocholine, and glycerine-phosphocholine (tCho), and total Glu and Gln (Glx) were quantified using LCModel (Version 6.3-1 L). We used the unsuppressed water spectrum as an internal reference instead of tCr, as tCr cannot be assumed to be stable (17). When comparing concentrations using a ratio, it can be challenging to determine which concentration is responsible for the observed difference (31). The possible impact of edema was evaluated in both preoperative and postoperative settings using T2-weighted images. No signs of edema were observed either preoperatively or at the time of postoperative MRS acquisition.

### Statistical analysis

Metabolites were assessed for normal distribution using the Shapiro-Wilks test and all the analyzed metabolites were normally distributed.

Preoperative concentrations of metabolites in patients were compared to those of a healthy control group using t-test. Changes in metabolite concentrations from preoperative to postoperative at the group level within the patient cohort were assessed using a paired t-test. Lateral differences between the left and right thalamus, and between the left and right lentiform nucleus, were similarly evaluated using paired statistics. As no lateral asymmetry could be established, the voxels in the respective anatomical regions were averaged and a separate t-test was performed for each metabolite concentration (tCr, tNA, tCho, Glx) in each averaged voxel representation (thalamus or lentiform nucleus). The LED (levodopa equivalent dose) was calculated using the LED conversion formula (29) and preoperative vs. postoperative values was evaluated. For all analyses a *p* < 0.05 was considered significant. Statistics was calculated using SPSS ver 29 (IBM, USA).

## Results

In total, 22 electrodes were implanted in 11 patients with a mean PD duration of 9.4 years. Two patients were excluded from the postoperative analysis due to their inability to remain still in the MR scanner. The targets for these two excluded patients were located in Zi. Consequently, all the remaining patients received bilateral electrodes in the STN. Due to unknown inherent errors in the stored dataset, one patient was excluded from the paired analysis because the preoperative examination was unreadable. One postoperative left thalamus and one postoperative left lentiform nucleus were discarded after quality inspection of the fitted spectra by an experienced MR physicist. Finally, one HC left thalamus was excluded due to voxel misplacement in the posterior direction. All other healthy, preoperative and postoperative spectra passed the quality inspection, and the corresponding determined concentrations were included in the subsequent analyses.

All patients were evaluated postoperatively in the neurological clinical practice and they all responded well with reported symptom relief. The positive clinical effect after surgery is supported by the significantly altered level of LED after surgery, see Table 1. The patients reduced their medication on group level with a preop of 925 ± 272 and a postop of 611 ± 210 (mean ± SD) LED. This represents a 32% reduction after surgery (*p* < 0.001). Importantly, when only including the patients with bilateral STN stimulation (two patients were operated in Zi and were unable to undergo the postoperative MRS) the postoperative reduction was estimated to 38%.

### Metabolite concentration changes

**Figure 2:**
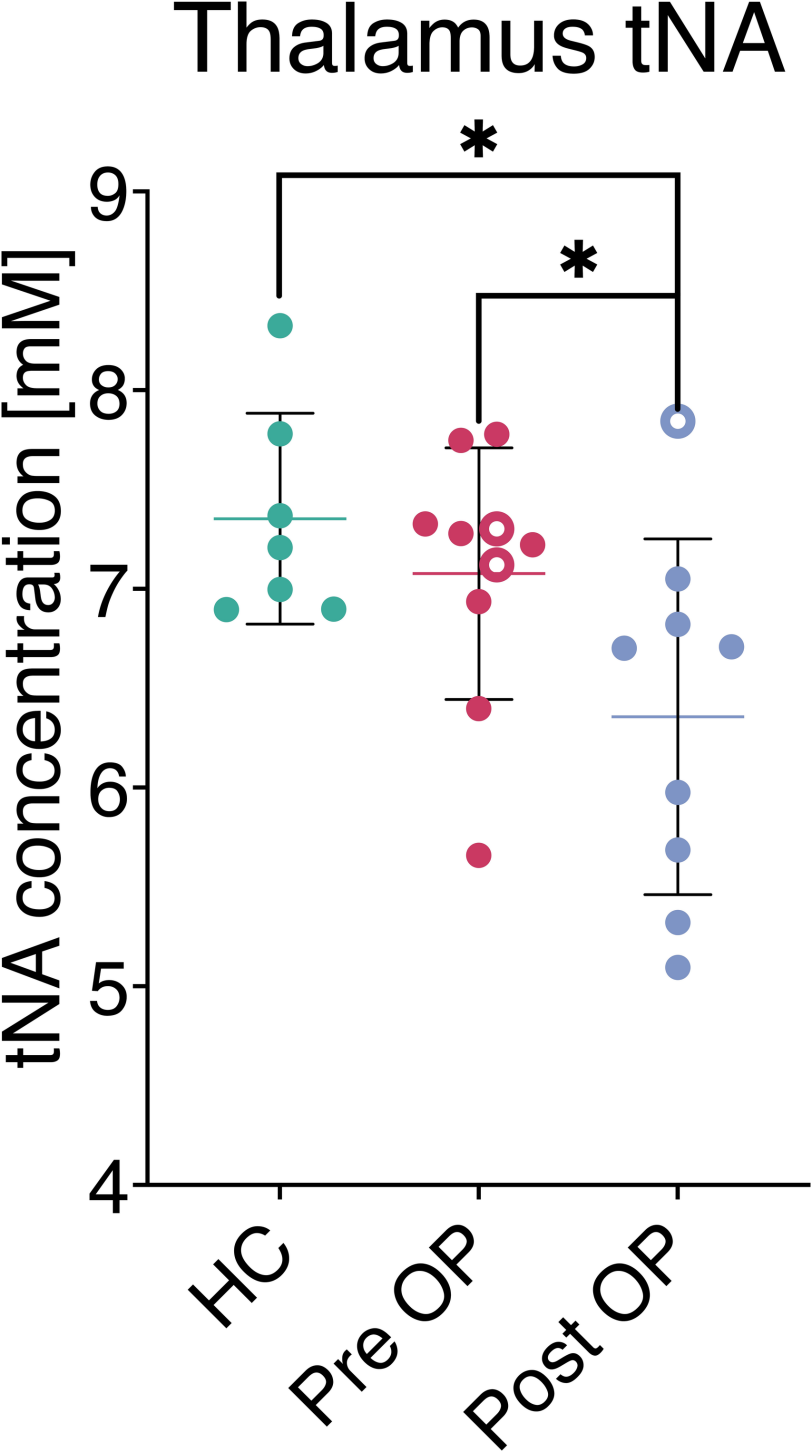
Metabolite concentration in thalamus (tNA; N-acetylaspartate and N-acetyl-aspartyl glutamate), measured in healthy control, preoperative and postoperatively. A significant change in tNA was observed following DBS, in comparison to both preoperative (*p* = 0.03) and healthy control (*p* = 0.02) concentrations. Missing paired measurements are represented as hallow rings; two patients lacked postoperative data, and one patient lacked preoperative measurement.

In patients scheduled for DBS surgery, compared to a healthy control group, no differences were found in the resulting metabolite concentrations (tNA, tCr, tCho, Glx) in the voxels placed in the thalamus and lentiform nucleus. In contrast, thalamic tNA concentrations differed significantly (*p* = 0.03) in the patient group when comparing postoperative 6.36 ± 0.90 mM and preoperative 7.08 ± 0.63 mM (mean +-SD), (Table 2, Figure 2). This represents a 10.2% reduction in thalamic tNA levels. The postoperative measurement in thalamus was also significantly changed (*p* = 0.02) compared to healthy controls 7.35 ± 0.53 mM (mean ± SD), (Figure 2). The resulting concentrations for the remaining metabolites in the thalamus, as well as all metabolites in the lentiform nucleus, showed no changes after surgery. See the flowchart, Supplementary Figure 1. Effect sizes and confidence intervals are reported in Supplementary Table 2.

**Table 2:** The resulting means and standard deviations (SD) of metabolite concentrations in mM (tCr, tNA, tCho, Glx).^*^

Metabolite	HC		PD		PD Postop		HC vs. PD	PD vs. PD **Postop**
	Mean	±SD	Mean	±SD	Mean	±SD	*p*-value	*p*-value
Thalamus								
tCr	4.94	0.47	4.90	0.28	5.02	0.78	n.s.	n.s.
tNA	7.35	0.53	7.08	0.63	6.36	0.90	n.s.	0.03
tCho	1.48	0.20	1.37	0.24	1.25	0.27	n.s.	n.s.
Glx	8.56	0.51	8.58	0.86	8.31	2.42	n.s.	n.s.
Lentiform nucleus								
tCr	4.96	0.70	4.84	0.45	5.10	0.44	n.s.	n.s.
tNA	5.85	0.38	6.31	0.79	6.15	0.42	n.s.	n.s.
tCho	1.10	0.13	1.16	0.21	1.14	0.13	n.s.	n.s.
Glx	9.53	0.74	8.82	1.22	9.61	2.00	n.s.	n.s.

In the thalamus and lentiform nucleus in healthy controls and Parkinson patients preoperatively and postoperatively^**^. Significant concentration differences are shown in bold.

* tCr; total creatine and phosphocreatine, tNA; N-acetylaspartate and N-acetyl-aspartyl-glutamate, tCho; total choline, phosphocholine, and glycerophosphocholine, Glx; total Glu and Gln.

** Three patients were excluded from the postoperative analysis. One patient was excluded from the paired analysis due to an unreadable preoperative examination, and two were excluded because they were unable to remain still during the MRI scan.

## Discussion

The main finding was a difference in thalamic tNA concentration in PD patients following DBS surgery targeting the STN. This result presents new metabolite information in a highly selected patient group with PD using the noninvasive MRS technique. The therapeutic neuromodulatory effect of DBS remains debated (9), and a change in tNA may suggest an induced modulation of thalamic motor neuronal function following DBS treatment (17, 19). No additional differences in metabolite concentrations were observed between before and after surgery, in either the thalamus or lentiform nucleus. This result aligns with the previously reported reduction in tNA following DBS implantation targeting the VIM in patients with ET (28).

### PD and treatment

The need of higher doses for symptomatic control may lead to disabling motor fluctuations as the disease progresses. This advanced stage of PD indicates the need for additional treatments, which includes DBS (4). The concept of levodopa equivalent dose (LED) was introduced to compare the intensity of medications in patients on various antiparkinsonian drugs, based on their dopaminergic effects by using a LED conversion formula (29). All patients reported symptom relief in the postoperative neurological assessment, and overall, PD medication was reduced by 32%. Unlike STN-DBS, Zi-DBS did not result in a reduction in LED which is a finding consistent with the literature (14). Thus, when only including the patients with bilateral STN stimulation, the reduction in LED was estimated at 38%, which is comparable to previous reports (32). In this study, the reduction in LED clearly demonstrates both the effectiveness of stimulation and the clinical benefit of targeting the STN with DBS in patients with PD.

### MRS

MRS provides a non-invasive, *in vivo* biochemical view of the brain and may support clinical decision-making (15, 16).

The voxels were placed in the lentiform nucleus and thalamus, regions both implicated in the pathophysiology of PD (1, 11), both before and after surgery. Our results support the normal findings in the lentiform nucleus (22–25) and in thalamic metabolite concentrations (23, 27), which have now also been observed in the specific group of PD patients scheduled for DBS. The unchanged values in the globus pallidus previously reported following DBS (33) also align with our data, where the metabolite concentrations in the lentiform nucleus remained unchanged.

However, inconsistent findings regarding metabolite concentrations in subcortical structures have been reported in MRS studies on PD patients (20). These include a reduction of NAA/Cho in the lentiform nucleus (21) and thalamic metabolite alterations in tremor dominant PD, with reduced NAA/Cr (26). It is important to note that the reported results from using MRS in PD research vary depending on whether they are expressed as tCr-ratios or absolute concentrations (in mM). Moreover, some studies use anatomical terms that are collective names, such as the striatum (34), which consists of both the caudate nucleus and putamen, and the lentiform nucleus (21–25), which includes the globus pallidus interna and externa, as well as the putamen. The anatomical name of the region referred to in the literature may also be described similarly but nevertheless investigate different parts within the specified anatomy. The distinct roles of adjacent thalamic nuclei in sensory and motor function (11), also highlight the importance of comparable voxel placement and size when reviewing previous reports. In addition, the voxel does not exclusively encompass the designated anatomical region but often also includes adjacent structures due to its geometric shape and size. These varying partial volume effects may therefore give rise to heterogenic results in different reports.

However, it appears that the concentrations of investigated metabolites (tCr, tNA, tCho, Glx) do not serve as potential biomarkers in the thalamus and lentiform nucleus, either for aiding the diagnosis of advanced PD or for surgical decision-making. Furthermore, the metabolite concentrations following DBS in the area around the lentiform nucleus appear unaffected by stimulation. The novelty in this study lies in the observed change in thalamic tNA level following the electrode placements in the STN.

### tNA

The tNA signal represents a combination of N-acetyl-aspartate (NAA) and N-acetyl-aspartyl-glutamate (NAAG) and it is typically the most prominent spectral resonance in brain spectra. Due to significant spectral overlap between these two metabolites, it is difficult to differentiate their respective contribution to the combined resonance (35). The observed change in tNA concentration may therefore reflect either a shared effect or an underestimated change if a concurrent, smaller opposing effect occurred in one or the other metabolite. In the literature, the NAA value is often reported, but it usually refers to the tNA value, since the individual components are not separated (20).

NAA is found within neurons, including the dendrites and axons (36) and is mainly a neuron specific metabolite (18). Reduced concentrations have been associated to several brain pathologies associated with irreversible neuronal or axonal loss following brain trauma, ischemia, and neurodegenerative diseases (19). However, reduced NAA concentrations cannot be uniformly translated to neuronal integrity, as elevated NAA can be associated with intellectual disability (19), while depleted NAA levels have been observed without extensive loss of viable neuronal tissue (37).

NAA is synthesized from aspartate and acetyl coenzyme in an energy-dependent manner within the mitochondria of neurons, and may therefore reflect the integrity of neuronal mitochondrial metabolism and/or neuronal metabolic activity. NAA is also involved in myelinogenesis and has been suggested to function as an osmolyte (17). Finally, NAA serves as the precursor of NAAG (17, 19) which is a regulatory neurotransmitter that includes modulating glutamatergic transmission (19). NAA and NAAG levels can both alter in response to stimuli and normalize to baseline following cessation. This reaction can be reciprocal, as was demonstrated in response to visual stimuli, where NAA concentrations decreased, and NAAG increased correspondingly (38). The reversibility of tNA has also been described in multiple sclerosis (39) and epilepsy (40).

### DBS effect

The altered medication following DBS surgery in STN may theoretically resulted in unknown alterations of metabolite concentrations. Ratios of striatal NAA/Cho (34) and putaminal NAA/Cr (41) levels were normal when on medication but not in drug naïve patients (34, 41). However, this treatment effect was not observed in the lentiform nucleus, where normal concentrations of tNA, tCho, and tCr, as well as the Glx/Cr ratio, were reported both before and after apomorphine administration (24). Additionally, the metabolites in globus pallidus remained unchanged after successful DBS with bilateral implantation of electrodes in the STN (33). Importantly, none of the patients in this study where OFF medication during the MRS investigation and similar to our findings, reduced tNA concentrations have been observed with comparable thalamic voxel placement after DBS targeting the VIM in ET patients (28). PD and ET are distinct diseases with separate treatment regimens, which is why it seems more plausible that we have observed a DBS effect rather than the result of reduced LED.

As a result of the implantation, reduced tNA concentrations could be interpreted as permanent neuronal injury or a neurodegenerative progression. However, for both the previously reported ET cohort (28) and the current PD cohort, the reduction in tNA following surgery was approximately 10% (9.5% in ET and 10.2% in PD). The similar degree of change between cohorts, despite the lead being less included in the thalamic voxel in the PD group, suggests that potential microstructural damage from the lead accounts for the result only to a minor extent. Moreover, only a thin tissue reaction around the lead tract has been described (42, 43). Thus, considering the well-known reversible nature of DBS therapy and tNA levels, the reduction in tNA following surgery rather suggests a modulation of neuronal metabolic activity in the motor part of thalamus in response to stimulation.

### Limitations and strengths

The MRS-voxels were not as small or precise as would be desired due to technical limitations (examination times), which is why we use the collective term ‘*lentiform nucleus’* instead of referring to its separate anatomical structures. Furthermore, it is not possible to include only the region of interest in the voxel, as the motor part of the thalamus cannot be spatially isolated while entirely avoiding unwanted anatomy in the vicinity.

There are also subtle individual anatomical variations in brain anatomy, leading to different contributions from partial volume effects within the voxel. Furthermore, the MRS technique does not specifically differentiate between intra- or extra-cellular location, which may be a factor to consider when interpreting the metabolite values.

Patients selected for DBS surgery represent a distinct, well-defined group with advanced PD eligible for surgery. Additionally, the statistical analysis was intrinsically paired, meaning significant results should still be possible to establish, despite the limited size of the study population. However, if a larger group had been studied, more subtle metabolite differences may have been detected. Of note, the study population consisted entirely of Caucasian participants and therefore shows limited diversity. A limitation is the wider age range in the HC group, which may have introduced variability and age-related effects despite similar median ages. Furthermore, the results are exploratory and preliminary, and apply to patients undergoing DBS with STN-targeted electrodes, but not to the PD population as a whole.

Due to MR-safety conditions, the MRS was performed while the patients were OFF stimulation. Additional differences in metabolite concentrations might also have been possible to observe if the MRS had been conducted while stimulation was ON. Same-subject repeat measurement variability was not investigated. However, variability is unlikely to account for our findings due to the statistical methods employed. Importantly, the two patients with Zi electrodes were unable to undergo postoperative MRS due to intolerable tremor when stimulation was OFF. Therefore, the paired analysis showing the thalamic difference in tNA was only measured in patients with electrodes placed in the STN. No clinical rating scale was recorded; nevertheless, all patients responded well after surgery, according to postoperative evaluations in neurological clinical practice, with self-reported symptom relief. The clinical effectiveness of the DBS implantation was also evidenced by the overall reduction in LED medication postoperatively (see Table 1) with comparable results in the literature (32).

Due to significant spectral overlap between NAA and NAAG, it was difficult to differentiate between them, and the combined resonance of tNA was measured. Since the effect of the two compounds differ it would be of value to be able to measure the two separately. Nevertheless, our results suggest that both metabolites may have been decreased as a result of the therapy. Alternatively, we underestimated the relatively larger reduction of one of the metabolites in the case of a reciprocal reaction. Water-scaled concentrations were used to enable comparison before and after DBS, and MRS was performed in the chronic phase, when the edema associated with the perielectrode space (30) had already resolved. Importantly, since the other metabolites remained unaltered, potential magnetic distortion from the lead is unlikely. In addition, the lead did not appear to affect the spectral quality of the MRS measurements postoperatively.

Comparability of findings are influenced by heterogeneous patient groups, which may differ in both disease severity and therapeutic regimens (20). The tissue heterogeneity within the field of MRS can also influence the comparability of findings. However, the similar results obtained from the thalamic voxel, when compared to a previous study (28) that shares comparable voxel placement, size, acquisition parameters, and field strengths, not only strengthen the presented findings but also highlight the utility and relevance of MRS as a powerful research tool-especially when the analysis methods are consistent across studies.

### Future

Future technical advances in delineating voxel shape and reducing its size, along with the ability to conduct investigations with stimulation ON, will add value to the MRS method in the assessment of PD pathophysiology and DBS effects. Research involving larger study populations and the separation of NAA from NAAG resonances could also reveal more details about the induced alterations and uncover more subtle metabolite changes.

## Conclusion

This study provides novel insight into a DBS effect in the motor region of the thalamus on a metabolite level using MRS. The thalamic tNA concentrations in PD patients were normal compared to healthy controls before surgery, but was altered after DBS implantation targeting the STN. No other differences in metabolite levels were observed in thalamus or lentiform nucleus, when comparing PD patients, before and after surgery, to healthy controls.

## Data Availability

The datasets presented in this article are not readily available because the data that support the findings of this study are not publicly available due to patient confidentiality. Requests to access the datasets should be directed to peter.zsigmond@liu.se.
