# Supplementary material for: Thalamic metabolite changes after subthalamic nucleus deep brain stimulation in Parkinson’s disease: an exploratory magnetic resonance spectroscopy study
Source: Front Neurol. 2025 Dec 3;16:1662142. doi: 10.3389/fneur.2025.1662142 (PMC12708281; doi:10.3389/fneur.2025.1662142)
Supplement: Supplementary file 1 [file Table_1.docx]

| **Supplementary table 1.** Description of the PD patients. Age, sex, disease duration and medication. The patients significantly reduced their medication after surgery, with a 32% reduction on group level. | | | | | | |
| --- | --- | --- | --- | --- | --- | --- |
| Patient | Age | Sex | Disease duration | Pre OP LED* [mg] | Post OP LED* [mg] |  |
|  |  |  |  |  |  |  |
| 1 | 62 | M | 13 | 1425 | 1075 |  |
| 2 | 64 | M | 8 | 1075 | 450 |  |
| 3** | 64 | M | 12 | 575 | 550 |  |
| 4** | 68 | F | 9 | 600 | 600 |  |
| 5 | 65 | M | 9 | 1075 | 550 |  |
| 6 | 61 | M | 12 | 1025 | 850 |  |
| 7 | 63 | M | 8 | 925 | 450 |  |
| 8 | 62 | M | 9 | 975 | 700 |  |
| 9 | 56 | M | 6 | 925 | 650 |  |
| 10 | 65 | M | 7 | 500 | 300 |  |
| 11 | 65 | F | 10 | 1075 | 550 |  |
|  |  |  |  |  |  |  |
| Median | 64 | Mean | 9 | 925 | 611 | p<0.001 |
|  |  |  |  |  |  |  |
|  |  |  |  |  |  |  |
| Healthy control | Age | Sex |  |  |  |  |
|  |  |  |  |  |  |  |
| 1 | 77 | M |  |  |  |  |
| 2 | 42 | M |  |  |  |  |
| 3 | 65 | M |  |  |  |  |
| 4 | 55 | M |  |  |  |  |
| 5 | 66 | M |  |  |  |  |
| 6 | 67 | F |  |  |  |  |
| 7 | 66 | M |  |  |  |  |
|  |  |  |  |  |  |  |
| Median | 66 |  |  |  |  |  |
|  |  |  |  |  |  |  |
| *LED = Levodopa Equivalent Dose. | | | | | | |
| ** Patients with leads in the zona incerta (Zi); all remaining leads were placed in the subthalamic nucleus (STN)” | | | | | | |
