# Supplementary material for: Thalamic metabolite changes after subthalamic nucleus deep brain stimulation in Parkinson’s disease: an exploratory magnetic resonance spectroscopy study
Source: Front Neurol. 2025 Dec 3;16:1662142. doi: 10.3389/fneur.2025.1662142 (PMC12708281; doi:10.3389/fneur.2025.1662142)
Supplement: Supplementary file 2 [file Table_2.docx]

| **Supplementary table 2.** The resulting means and standard deviations (SD) of metabolite concentrations in mM (tCr, tNA, tCho, Glx)*. In the thalamus and lentiform nucleus in healthy controls and Parkinson patients preoperatively and postoperatively**. Significant concentration differences are shown in bold. | | | | | | | | | | | | | | | | |
| --- | --- | --- | --- | --- | --- | --- | --- | --- | --- | --- | --- | --- | --- | --- | --- | --- |
|  |  |  |  |  |  |  |  |  |  |  |  |  |  |  |  |  |
|  | HC | | PD | | PD Postop | |  | HC vs PD | | | |  | PD vs PD postop | | | |
| Metabolite | Mean | ±SD | Mean | ±SD | Mean | ±SD |  | Effect size | Confidence interval | | p-value |  | Effect size | Confidence interval | | p-value |
| Thalamus |  |  |  |  |  |  |  |  |  |  |  |  |  |  |  |  |
| tCr | 4.94 | 0.47 | 4.90 | 0.28 | 5.02 | 0.78 |  | 0.13 | -0.34 | 0.44 | n.s. |  | -0.07 | -0.76 | 0.65 | 0.86 |
| tNA | 7.35 | 0.53 | 7.08 | 0.63 | 6.36 | 0.90 |  | 0.49 | -0.34 | 0.95 | n.s. |  | **0.98** | **0.13** | **1.62** | **0.03** |
| tCho | 1.48 | 0.20 | 1.37 | 0.24 | 1.25 | 0.27 |  | 0.50 | -0.13 | 0.36 | n.s. |  | 0.44 | -0.12 | 0.38 | 0.25 |
| Glx | 8.56 | 0.51 | 8.58 | 0.86 | 8.31 | 2.42 |  | -0.34 | -0.80 | 0.75 | n.s. |  | 0.06 | -2.27 | 2.60 | 0.88 |
| Lentiform Nucleus |  |  |  |  |  |  |  |  |  |  |  |  |  |  |  |  |
| tCr | 4.96 | 0.70 | 4.84 | 0.45 | 5.10 | 0.44 |  | 0.21 | -0.48 | 0.71 | n.s. |  | -0.32 | -0.76 | 0.34 | 0.40 |
| tNA | 5.85 | 0.38 | 6.31 | 0.79 | 6.15 | 0.42 |  | -0.69 | -1.14 | 0.24 | n.s. |  | 0.42 | -0.31 | 0.91 | 0.28 |
| tCho | 1.10 | 0.13 | 1.16 | 0.21 | 1.14 | 0.13 |  | -0.32 | -0.25 | 0.14 | n.s. |  | 0.50 | -0.05 | 0.19 | 0.20 |
| Glx | 9.53 | 0.74 | 8.82 | 1.22 | 9.61 | 2.00 |  | 0.68 | -0.39 | 1.82 | n.s. |  | -0.50 | -2.49 | 0.63 | 0.20 |
|  |  |  |  |  |  |  |  |  |  |  |  |  |  |  |  |  |
| *tCr; total creatine and phosphocreatine, tNA; N-acetylaspartate and N-acetyl-aspartyl-glutamate, tCho; total choline, phosphocholine, and glycerophosphocholine, Glx; total Glu and Gln. | | | | | | | | | | | | | | | | |
| **Three patients were excluded from the postoperative analysis. One patient was excluded from the paired analysis due to an unreadable preoperative examination, and two were excluded because they were unable to remain still during the MRI scan. | | | | | | | | | | | | | | | | |
