# Supplementary figures and images for: Thalamic metabolite changes after subthalamic nucleus deep brain stimulation in Parkinson’s disease: an exploratory magnetic resonance spectroscopy study
Source: Front Neurol. 2025 Dec 3;16:1662142. doi: 10.3389/fneur.2025.1662142 (PMC12708281; doi:10.3389/fneur.2025.1662142)

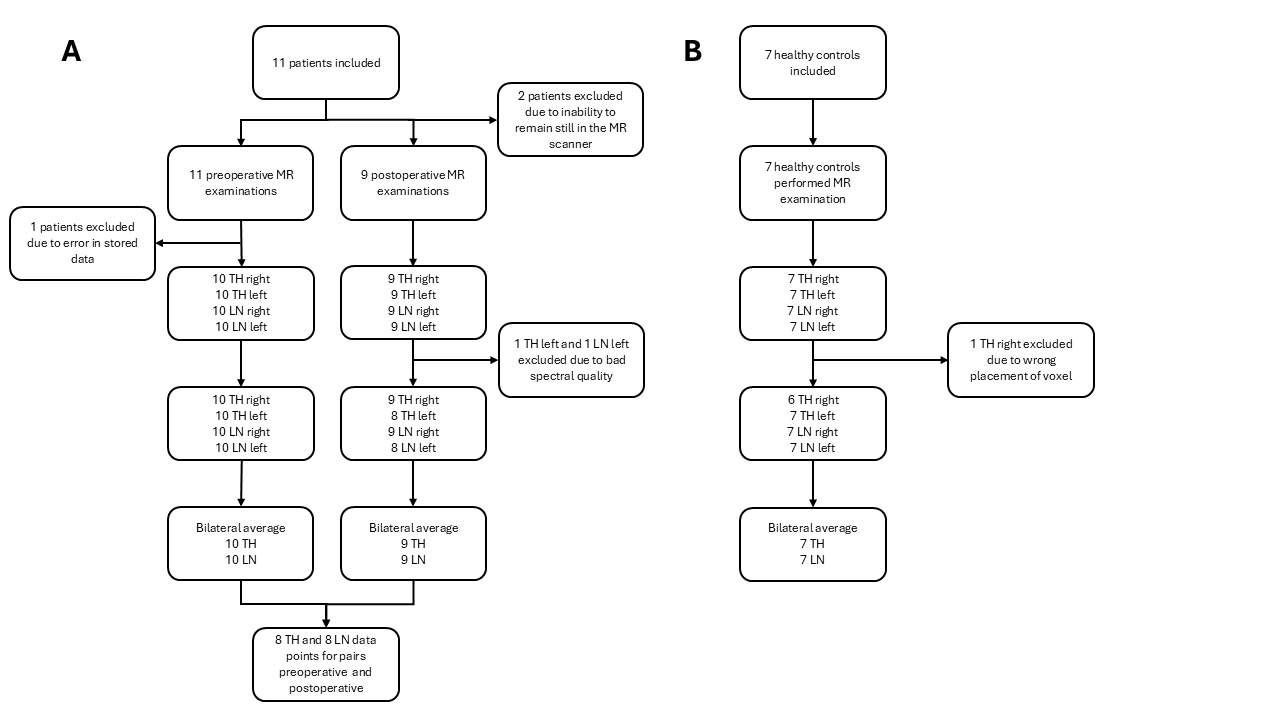

Supplement: Supplementary file 3 [file Image_1.png]
